# The Passion Principle

**DOI:** 10.1371/journal.pbio.1001629

**Published:** 2013-08-13

**Authors:** Daniel Simberloff

**Affiliations:** Department of Ecology and Evolutionary Biology, University of Tennessee, Knoxville, Tennessee, United States of America

## Abstract

Daniel Simberloff reviews E.O. Wilson's *Letters to a Young Scientist.*

Several decades ago, as a young scientist, I often received advice from a not-much-older Edward O. Wilson, especially during many long days of field work on small mangrove islands in Florida Bay. Some of it was direct and explicit—what to do, what not to do. But most of the advice was indirect, delivered during riveting discussions about what scientific discoveries and developments really advanced a field and why some disciplines seemed to advance more rapidly than others, or about personal squabbles that may have even retarded science a bit, and certainly did not advance it.

**Figure:**
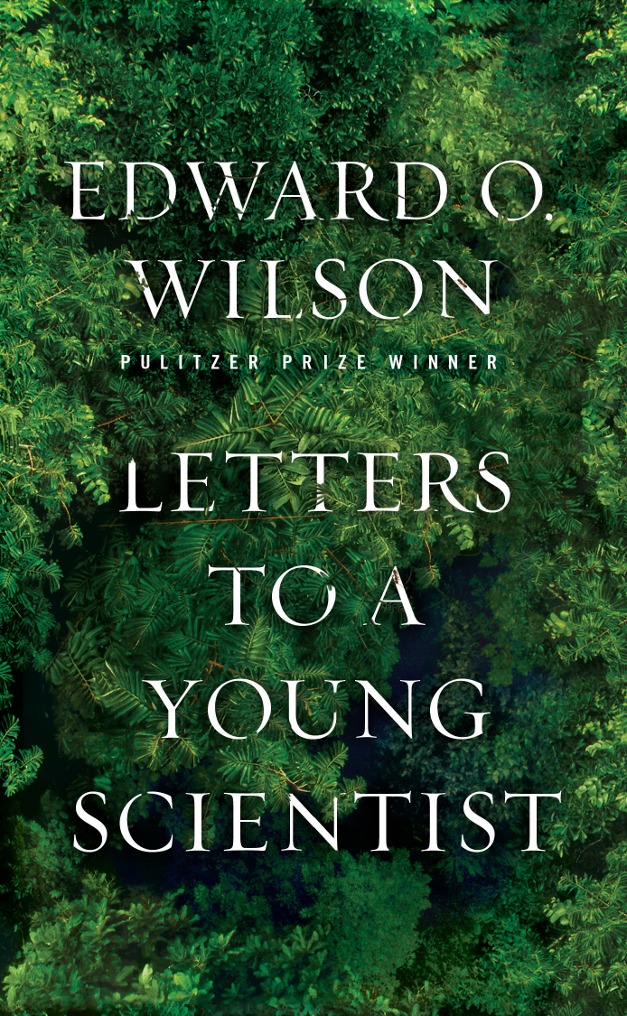
Wilson EO (2013) Letters to a Young Scientist. **New York: Liveright (W.W. Norton). 245 p. ISBN 978-0871403773 (hardcover). US$21.95.**

Now, as an old scientist, I am fascinated reading 20 letters that Wilson has penned specifically for young scientists. In many ways he has been remarkably consistent with what he told me nearly 50 years ago in Florida Bay. The letters, each a chapter primarily addressing a specific topic, draw heavily from Wilson's own remarkable trajectory from a young boy in Alabama obsessed with snakes and black widow spiders to one of the world's leading biologists with enormous contributions in ecology, evolution, myrmecology, behavior, conservation, and biogeography. Though the book is ostensibly for young people, not-so-young people will also enjoy it and find much inspiration. Some chapters are clearly aimed at people about Wilson's age when he was exploring Alabama swamps in his quest to become an Eagle Scout. Others seem to target graduate students, post-doctoral fellows, and beginning faculty. Yet the book, with a style that transcends the “young adult” genre, will engage any scientist. In fact, because of the autobiographical material, the thoughtful insights, and a few bombshells Wilson detonates along with way, this book is difficult to put down.

Those who have read his autobiography, *Naturalist* [1], will recognize some of the vignettes Wilson has chosen as vehicles for his advice. But even for stories that are well-known, he presents new interesting details and often enlightening context. Wilson as a child chasing butterflies in Washington, D.C. and Alabama. Wilson as a teenage herpetologist teaching Boy Scouts how to handle (or, as it were, how not to handle) venomous snakes. Wilson as a young ant enthusiast on the trail of the large primitive ant *Daceton armigerum* in Surinam—these or any of several other short narratives all give some insight into the underlying passion that he sees as the single most crucial attribute of a good scientist. Find the passion that grips you and follow it, no matter training or lack of it, and don't worry if it seems out of vogue.

A second piece of advice is perhaps more surprising—seek some area of science that is not in fashion. “If a subject already is receiving a great deal of attention…stay away from the subject.” He formalizes this as Principle Number Three: “March away from the sound of guns. Observe the fray from a distance, and while you are at it, consider making your own fray.” When Wilson discovered ants, not many myrmecologists occupied the scene. Today myrmecology might not be a great choice, according to Principle Number Three, but one presumes that sufficient passion and creativity can allow an exciting, rewarding career even in such a crowded field—as witness Corrie Saux Moreau, Wilson's last myrmecological doctoral student. He uses Moreau (now Assistant Curator at the Field Museum and a leading expert on ant systematics) to exemplify another trait that he sees as necessary for a successful career—enough self-confidence in one's own ideas to persevere in the face of obstacles and skepticism that others might put in your way.

Less controversial might be Principle Number Five: “For every problem in a given discipline of science, there exists a species or other entity or phenomenon ideal for its solution.” For Wilson, the red imported fire ant *(Solenopsis invicta)* exemplifies this principle. His research on its behavior led Wilson to discoveries that helped shape the entire discipline of sociobiology and made the species a classic case study in chemical ecology. However, another part of Principle Number Five is more surprising: “For every species or other entity or phenomenon, there exist important problems for the solution of which it is ideally suited.” Wilson relates this aspect of the principle to the fact that, of several million species on earth, at most a few dozen “model species” are really well-known, and he argues that studying an organism one loves intensely will likely yield interesting discoveries; it is the underlying passion that counts. This is exactly what he repeatedly told me as we rocked in small boats or scrambled over mangrove islands in Florida Bay so many years ago.

Many of Wilson's admonitions are not at such a grand scale but are very practical; this is particularly true of advice pitched to young faculty. I (and many of my colleagues) certainly agree with “Avoid department-level administration beyond thesis committee chairmanships if at all fair and possible. Make excuses, dodge, plead, trade…Consider carefully job offers from other universities or research institutions that include more research time and fewer teaching and administrative responsibilities.” Probably many field ecologists, at least, would agree with “Real scientists do not take vacations. They take field trips or temporary research fellowships in other institutions,” but maybe they would not admit this to their partners or children.

So far the various recommendations are in line with those a younger Wilson offered to a younger me; I listened, and they have served me well. But one piece of advice is rather different from the rest, or at least presented in a way and with such insistence that the message comes across differently. This message, and an essay based on this part of the book published in the *Wall Street Journal* [2], have aroused much controversy. Wilson, a courtly and genuinely kind person, seems to go through his scientific life generating greater (think sociobiology) or lesser (think group selection) controversies, and he has done it again. In the 1960s, Wilson felt so strongly about the importance of mathematics to biology that, as a faculty member, he sat through undergraduate math courses to remedy his weak college and grad school math background. And he advised me at that time to continue to pursue mathematics, including computer simulations, on the grounds that mathematics had as much to offer biology as chemistry did, and it was an under-explored nexus (consistent with Principle Number Three).

Yet beginning with the second letter—“Mathematics”—Wilson is at pains to tell at least budding biologists that, if they cannot or do not want to learn mathematics, not to worry, it will not be an impediment to a gratifying and important scientific career. It is this advice that has led to severe overt criticism in such public media as the the *Huffington Post* and *Slate* and to feverish concern among theoretical biologists in blogs, in discussion groups, and around proverbial water coolers. A close, calm read makes it clear that Wilson, in fact, did not write that math is unimportant to biology, or to science. He is explicit that in certain sciences (e.g., much of physics and chemistry) facility in advanced math is a sine qua non. What Wilson did write is that many great advances in biology can be achieved with fairly basic mathematics and that, if one wishes to tackle a problem requiring greater mathematical skill than he or she possesses, it is far more efficient to collaborate with a mathematician or math-minded biological colleague than to struggle to master the requisite math oneself. He even exemplifies this notion with some of his own fruitful collaborations with mathematicians such as George Oster and William Bossert and with biologists such as (notably) Robert MacArthur and (less notably) me. In fact, Wilson codifies his point here in Principle Number Two: “For every scientist, whether researcher, technologist, or teacher, of whatever competence in mathematics, there exists a discipline in science for which that level of mathematical competence is enough to achieve excellence.”

It is possible that mathematical theorists are particularly thin-skinned and would have reacted vehemently no matter how Wilson had phrased this message. However, the impact is greater by virtue of his repeating it, sometimes in less nuanced terms, at several points in the book and because of his luminous stature in biology. In addition, some gratuitous remarks seem almost designed to raise hackles: “The annals of theoretical biology are clogged with mathematical models that either can be safely ignored or, that when tested, fail. Possibly no more than 10% have any lasting value.” There is little doubt that many mathematical models had little lasting value and can now be ignored, though it is possible that some served a useful role in inspiring further thinking and data-gathering before being discarded. However, the statement and estimate are not really germane to Principle Number Two, which is convincing based on the rest of this letter and other letters in the book and an appreciation of Wilson's accomplishments and modus operandi.

A letter on “Most Likely to Succeed” raises another contentious issue, “groupthink,” the notion that bringing people together, especially people of somewhat different backgrounds, to mull over a scientific problem or concept is likely to lead to creative thinking. Wilson suggests that, in his experience, the more creative thinking is usually associated with individuals, not groups, and that these individuals are often loners, oddballs, antiauthoritarians, introverts, and not voted most likely to succeed. Wilson sees the role for groups of collaborators as much later in the process, after the idea has “hatched” and it is evident that various kinds of skills may be needed to develop it further or to test it.

Throughout the letters, Wilson clearly is thinking primarily of biologists, and probably biologists working at the individual level or above—communities and ecosystems. He strives to address all scientists and adds physical sciences to life sciences in various lists, but by virtue of his life's work and his longstanding interest in biogeography and in the various unstudied aspects of the earth's biodiversity, he tends to focus heavily on examples and scenarios that ring true to evolutionary ecologists and students of various taxa. Mutatis mutandis, some apply well to other sciences. Others may transfer less readily. Nevertheless, any young scientist would gain a lot from reading this book, and even old scientists will find much to ponder, both in relating Wilson's dicta to their own evolution as scientists and in how they train the next generation.

**About the Author**

Daniel Simberloff is Nancy Gore Hunger Professor of Environmental Studies at the University of Tennessee. He studies ecological and evolutionary questions and is especially interested in the causes and consequences of biological invasions. His new book Invasive Species: What Everyone Needs to Know (2013) follows The Encyclopedia of Biological Invasions (2011), edited with Marcel Rejmánek. His website is http://eeb.bio.utk.edu/peopletwo/daniel-simberloff/.
